# The BUB1 and BUBR1 paralogs scaffold the kinetochore fibrous corona

**DOI:** 10.1126/sciadv.ady6890

**Published:** 2025-09-12

**Authors:** Verena Cmentowski, Andrea Musacchio

**Affiliations:** ^1^ Department of Mechanistic Cell Biology, Max Planck Institute of Molecular Physiology, Dortmund 44227, Germany.; ^2^ Centre for Medical Biotechnology, Faculty of Biology, University of Duisburg-Essen, Essen 45141, Germany.

## Abstract

The kinetochore corona, a polymeric fibrous structure, facilitates chromosome biorientation and mitotic checkpoint signaling during mitosis. How its main building block, the ROD-Zwilch-ZW10 (RZZ) complex, assembles on the outer kinetochore remains poorly understood. Harnessing corona biochemical reconstitutions and cell biology, we reveal that the paralogous spindle assembly checkpoint (SAC) proteins BUB1 and BUBR1 promote nonredundant branches of corona assembly. MPS1 kinase–dependent kinetochore docking of BUB1 and subsequent recruitment of BUBR1 initiates assembly. Disrupting the first branch by depleting CENP-E, a kinesin that links BUBR1 to RZZ, uncovered a second assembly pathway mediated by a direct interaction between BUB1 and ROD. Discovery of a direct interaction with the RZZ explains how the SAC protein MAD1 fits this corona assembly scheme. Our findings solve the long-standing puzzle of corona assembly and demonstrate the intimate interweaving of chromosome biorientation and checkpoint signaling.

## Teaser

The kinetochore fibrous corona docks on CENP-E and a second elusive receptor identified as the checkpoint protein BUB1.

## INTRODUCTION

Kinetochores connect chromosomes to spindle microtubules and are essential for chromosome biorientation, mitotic fidelity, and genome stability (*1*, *2*). Kinetochores are made of >80 different polypeptides with scaffolding and regulatory functions. The kinetochore corona is a crescent-shaped fibrous structure (*3*). It assembles beyond the KMN (Knl1 complex-Mis12 complex-Ndc80 complex), the main component of the microtubule-binding outer kinetochore layer. Early in mitosis, the corona transports chromosomes as cargoes toward the spindle equator using dynein-dynactin (DD) and CENP-E, microtubule motors of opposite polarity (*4*). Concomitantly, the corona promotes spindle assembly checkpoint (SAC) signaling by docking the mitotic arrest deficient 1:mitotic arrest deficient 2 (MAD1:MAD2) complex (*3*, *5*). When Ndc80C-dependent end-on microtubule attachments replace motor-based lateral interactions, the corona disassembles, silencing the SAC and licensing sister chromatin separation (*6*–*8*). CENP-E, the ROD-Zwilch-ZW10 (RZZ) complex, and the DD adaptor Spindly are the corona building blocks. Various outer kinetochore factors partially required for corona stability have been previously identified, but a comprehensive molecular view of corona assembly is missing. Using biochemical reconstitutions, electroporation of recombinant proteins, and separation of function mutants, we now describe the entire corona assembly pathway. Our findings reveal that a complex of the paralogous SAC proteins BUB1 and BUBR1 initiates all interactions required for corona assembly. This resolves a long-standing question and underscores why chromosome biorientation and SAC signaling should be viewed as integrated and inseparable phenomena.

## RESULTS

### KNL1 scaffolds corona assembly

**Fig. 1.**
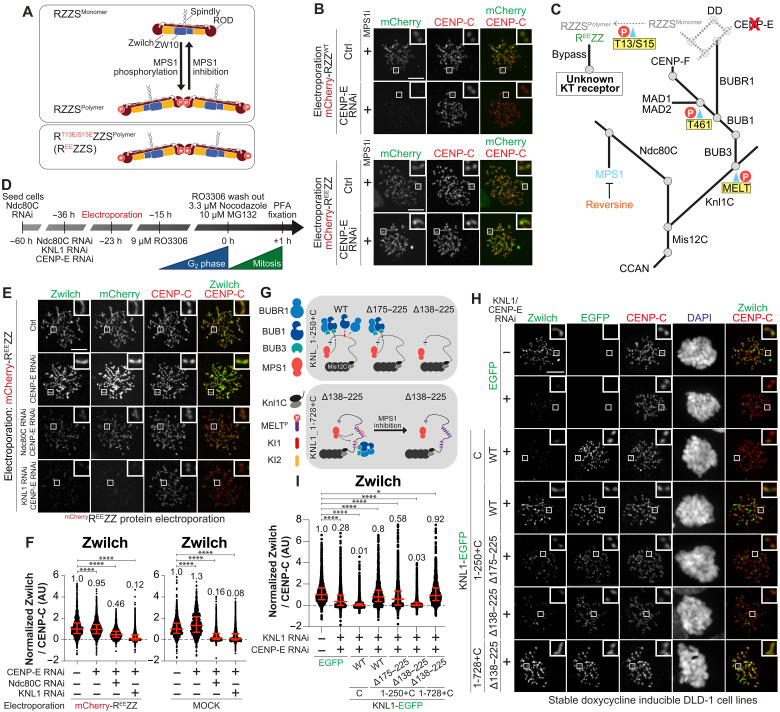
KNL1 promotes corona recruitment. (**A**) MPS1 phosphorylation–dependent polymerization of RZZS. (**B**) Images of electroporated mCherry-RZZ^WT^ or mCherry-R^EE^ZZ in CENP-E RNAi cells. Cells were synchronized in G_2_ (RO3306, 15 hours), released into mitosis in 3.3 μM nocodazole, 10 μM MG132, and 500 nM reversine (1 hour) before fixation. CENP-C visualizes kinetochores. Scale bar, 5 μm. Experiment performed three times. (**C**) Outer kinetochore organization. Straight lines represent different proteins or complexes, and gray circles direct protein interactions. Residues in yellow are MPS1-phosphorylated. Bypass conditions in (B) reveal existence of second RZZ receptor. (**D**) Scheme for cell synchronization and RNAi treatment for experiments in (E). h, hours. (**E**) Zwilch localization in cells electroporated with mCherry-R^EE^ZZ. Scale bar, 5 μm (**F**) Quantification of Zwilch levels in (E). *n*, individually measured kinetochores; (), cells [left to right: *n* = 1655 (34), 1775 (29), 2451 (39), 2730 (45), 2078 (41), 1148 (32), 1032 (29), and 2735 (41)]. Experiment performed three times. (**G**) Scheme of MPS1-dependent recruitment of BUB1/BUB3 and BUBR1/BUB3 to KNL1 constructs in (H). (**H**) Zwilch localization in stable DLD-1 cells expressing KNL1. Expression was induced with doxycycline 24 hours after seeding and KNL1/CENP-E depletion. Cells were synchronized in G_2_ with RO3306 (15 hours), released, and immediately treated with 3.3 μM nocodazole, 10 μM MG132, and doxycycline (1 hour) before fixation. DAPI stains DNA. Scale bar, 5 μm. (**I**) Zwilch quantification for (H). Left to right: *n* = 1576 (33), 1946 (42), 2556 (50), 1772 (36), 1951 (45), 1647 (38), and 1272 (32). Experiment performed three times. Statistical analyses (F and I) were performed with a nonparametric *t* test comparing two unpaired groups (Mann-Whitney test). *P ≤ 0.05, ****P ≤ 0.0001. Red bars are median (value above scatter dot plot) and interquartile range of normalized kinetochore intensities.

Assembly of the corona into a fibrous meshwork arises from polymerization of the RZZ complex and also requires binding of Spindly to the RZZ (forming the RZZS complex) and MPS1 kinase–mediated phosphorylation of threonine 13 (T13) and serine 15 (S15) on the ROD subunit (*9*–*11*) (Fig. 1A). We have recently reported that when MPS1 kinase is inhibited with reversine (*12*) to prevent RZZ polymerization, CENP-E becomes essential for kinetochore recruitment of RZZS (*13*). This, and additional evidence, identifies a previously unrecognized role of CENP-E in corona assembly and disassembly (*14*–*16*). Electroporation of the 812-kDa recombinant RZZ hexamer (tagged with mCherry) did not rescue corona assembly in cells depleted of CENP-E and treated with reversine, but electroporation of the equivalent phosphomimetic T13E-S15E (R^EE^ZZ) mutant complex did (Fig. 1B) (*13*). Thus, the corona assembles on at least one other receptor in addition to CENP-E.

To identify the elusive receptor, we initially assessed how the outer kinetochore affects RZZ recruitment (see schematic in Fig. 1C). Simultaneous depletion of the Ndc80 complex (Ndc80C) and of KNL1 by RNAi (directed against multiple subunits in the case of Ndc80C, see Materials and Methods) eliminated RZZ entirely, while individual depletions of KNL1 or Ndc80C caused strong depletion, rather than elimination, of kinetochore-bound RZZ (fig. S1, A to D). These experiments implicate Ndc80C and KNL1 as being upstream of RZZ recruitment, but do not imply that they have a direct role. First, Ndc80C and KNL1 sustain each other reciprocally at the kinetochore (quantified in fig. S1, C and D) (*17*). Second, each promotes recruitment of other proteins involved in RZZ recruitment (Fig. 1C). Specifically, Ndc80C is essential for kinetochore recruitment of MPS1 kinase (*18*–*21*), which promotes RZZS oligomerization (Fig. 1, A and C). Conversely, KNL1 is necessary for the recruitment of ZWINT (the second subunit of the Knl1C complex) and BUB1, which is itself MPS1 dependent (*10*, *22*–*30*). Furthermore, BUB1 recruits the MAD1:MAD2 complex (also through MPS1), CENP-F, and BUBR1, whose pseudokinase domain promotes CENP-E recruitment, thereby indirectly contributing to localization of the RZZ complex to the kinetochore (Fig. 1C) (*13*, *31*). Depletion of Ndc80C and KNL1 abrogated kinetochore localization of BUB1 and CENP-E in addition to RZZ (fig. S1, E and F).

Because mCherry-R^EE^ZZ localizes to kinetochores independently of CENP-E and MPS1 activity, monitoring its requirements for localization might enable us to close in on the RZZ receptor. We electroporated mCherry-R^EE^ZZ into cells depleted of either KNL1 or Ndc80C and monitored its localization with and without MPS1 inhibition (Fig. 1D). As CENP-E is not required for mCherry-R^EE^ZZ localization, we took the precaution of codepleting CENP-E in these experiments to avoid possible confounding effects caused by its role in kinetochore recruitment of endogenous RZZ (fig. S1J). Codepletion of Ndc80C and CENP-E permitted substantial residual mCherry-R^EE^ZZ localization (Fig. 1, E and F). Further addition of reversine eliminated residual mCherry-R^EE^ZZ (fig. S1, G to I), indicating that elimination of Ndc80C exacerbates the consequences of MPS1 inhibition by reversine, in line with the role of Ndc80C in recruiting MPS1 (*20*, *21*, *32*). As mCherry-R^EE^ZZ bypasses MPS1 in RZZS polymerization, this observation suggests that MPS1 activity is required for an additional aspect of RZZS recruitment. Contrary to the codepletion of Ndc80C and CENP-E, codepletion of KNL1 and CENP-E prevented mCherry-R^EE^ZZ recruitment very robustly, and further inhibition of MPS1 had only a minor additional effect (Fig. 1, E and F, and fig. S1, G to I).

Collectively, these experiments point to a crucial role of the KNL1 axis in RZZ recruitment, facilitated by MPS1 kinase activity. To identify regions of the 2316-residue KNL1 protein (isoform 2; fig. S2A) relevant to RZZ recruitment, we depleted KNL1 and CENP-E and expressed different KNL1 constructs fused to an EGFP tag in stable DLD-1 cell lines (Fig. 1, G to I, and fig. S2, C and D). This analysis revealed that constructs containing at least 1 of the 19 MELT (Met-Glu-Leu-Thr) motifs of KNL1 rescued recruitment of RZZ to kinetochores. As MELT motifs bind BUB1:BUB3 upon phosphorylation by MPS1 (*24*, *27*–*31*, *33*, *34*) (Fig. 1, G to I, and fig. S2, B and E), this observation argues that BUB1, or a protein downstream of BUB1, contributes to kinetochore recruitment of RZZS in addition to allowing CENP-E recruitment through BUBR1.

**Fig. 2.**
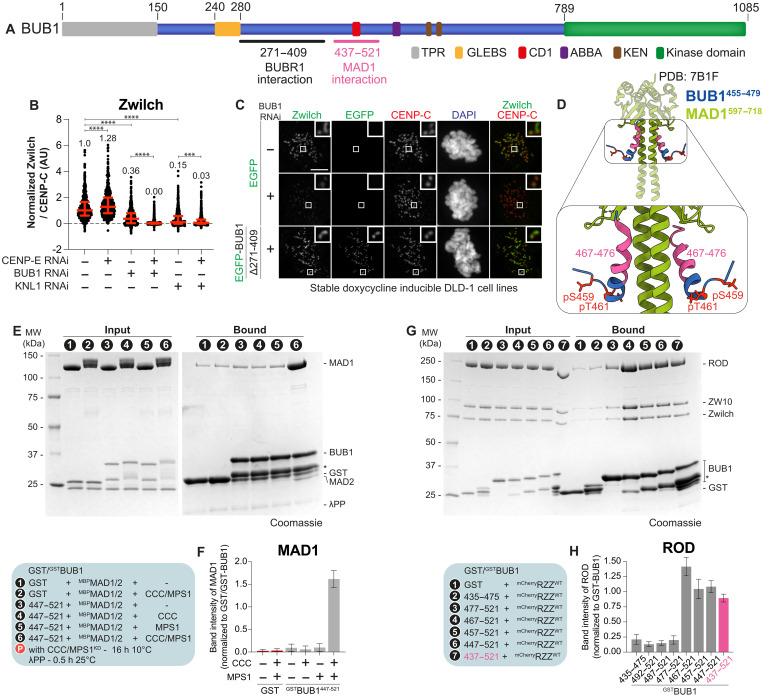
RZZ and MAD1 bind directly to an overlapping region of BUB1. (**A**) Functional domains of BUB1. (**B**) Zwilch quantification in DLD-1 cells with indicated depletions. Twenty-four hours after seeding and siRNA treatment, cells were synchronized in G_2_ (9 μM RO3306, 15 hours) and released into mitosis in 3.3 μM nocodazole and 10 μM MG132 (1 hour) before fixation. *n*, individually measured kinetochores; (), cells [left to right: *n* = 662 (29), 541 (24), 748 (26), 916 (28), 803 (27), and 778 (25)]. Experiment performed two times. (**C**) Zwilch localization in stable DLD-1 cells expressing indicated BUB1 constructs. Twenty-four hours after seeding and BUB1/CENP-E siRNA treatment, doxycycline was added and cells were synchronized in G_2_ phase (RO3306, 15 hours). Cells were released into mitosis in 3.3 μM nocodazole, 10 μM MG132, and doxycycline (1 hour) before fixation. CENP-C/DAPI visualize kinetochores/DNA. Scale bar, 5 μm. Experiment performed three times. (**D**) Structure of BUB1^455-479^ and MAD1^597-718^ (PDB ID: 7B1F) (*43*). (**E**) SDS-PAGE of pulldown assay with indicated GST fusions baits and MBP-MAD1/2 prey. Bait and prey were incubated with CCC and MPS1^KD^ (16 hours, 10°C). Eluates were dephosphorylated with λ-phosphatase (30 min, 25°C) before SDS-PAGE. Asterisk: contaminants/degradation products. (**F**) MAD1 band intensity normalized to bait from experiment in (E). Mean and SD from three independent experiments. (**G**) SDS-PAGE of pulldowns with indicated GST baits and mCherry-RZZ prey. Asterisk: contaminants/degradation products. (**H**) ROD band intensity normalized to bait from (G). Mean and SD from three independent experiments. Statistical analysis in (B) was performed with a nonparametric *t* test comparing two unpaired groups (Mann-Whitney test). ***P ≤ 0.001, ****P ≤ 0.0001. Red bars represent the median (value shown above scatter dot plot) and interquartile range of normalized kinetochore intensities.

To investigate this question, we depleted BUB1 (whose organization is schematically shown in Fig. 2A) by RNAi and assessed the impact on RZZ. In comparison to KNL1 depletion, which caused a very strong decrease of kinetochore RZZ, or to simultaneous depletion of CENP-E and BUB1, which completely removed RZZ from kinetochores (quantified in Fig. 2B), BUB1 knockdown only caused an incomplete depletion of RZZ from kinetochores (quantified in Fig. 2B). These results may seem inconsistent with the known epistatic relationships between the depleted proteins, as depletions of (i) KNL1, (ii) BUB1, and (iii) BUB1 and CENP-E ought to phenocopy each other with regard to RZZ recruitment (Fig. 1C). Nonetheless, previous studies investigating the involvement of BUB1 in RZZ recruitment have also yielded conflicting conclusions (*26*, *35*–*41*), possibly due to incomplete depletions or ectopic interactions (as discussed in the Discussion). Thus, we opted to continue investigating whether BUB1, or a protein downstream of BUB1, provides a platform for RZZ docking in addition to that provided by the BUBR1-CENP-E axis. Because CENP-E is dispensable for RZZ recruitment when RZZ forms polymers, we limited the analysis to the three proteins known to directly require BUB1 for kinetochore recruitment: CENP-F, BUBR1, and MAD1:MAD2. We have already shown that CENP-F is dispensable for RZZS recruitment (*42*). To test a CENP-E–independent role of BUBR1, we depleted BUB1 and replaced it with a BUB1 transgene lacking the BUBR1-binding region (∆271 to 409) (Fig. 2A). This mutant continued to recruit RZZS (Fig. 2C), thus leaving only MAD1 or BUB1 itself as potential RZZ receptors.

### BUB1 binds directly to RZZ

The C-terminal domain of MAD1 (MAD1^CTD^, residues 585 to 718) binds directly to residues 455 to 479 of BUB1 after phosphorylation of residues Ser^459^ and Thr^461^ by CDK1 and MPS1, respectively (Fig. 2D) (*43*, *44*). Deletion of residues 437 to 521 of BUB1 encompassing this MAD1 binding site was also shown to reduce RZZ kinetochore levels (*36*). Previous evidence, discussed below, suggests that RZZ acts upstream of MAD1:MAD2 recruitment. On the basis of the observations described here, we hypothesized that BUB1, by binding MAD1, may also facilitate RZZ recruitment. To test this, we first determined whether we could reconstitute binding of MAD1:MAD2 to an immobilized glutathione *S*-transferase (GST)–tagged fragment of BUB1 encompassing the previously identified binding region (BUB1^447-521^). Full-length MAD1:MAD2 bound robustly to this fragment of BUB1 after in vitro phosphorylation with MPS1 and CDK1, and preventing phosphorylation abrogated binding (Fig. 2, E and F, and figs. S3A and S5, A and B), in line with a previous report (*43*).

**Fig. 3.**
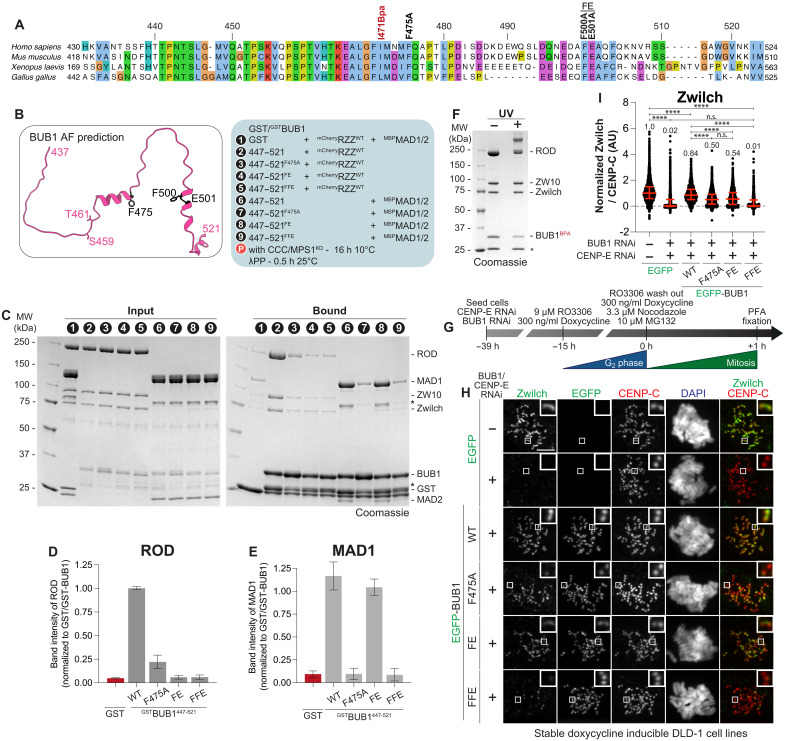
Validation of the RZZ-binding site of BUB1. (**A**) Multiple sequence alignment of the BUB1 central domain, with mutations discussed in the main text indicated above. (**B**) AlphaFold prediction of BUB1^437-521^ (AF-O43683-F1-v4, https://alphafold.ebi.ac.uk/entry/O43683). Amino acid substitutions are highlighted in black. (**C**) SDS-PAGE of a pulldown assay with either GST or GST-tagged BUB1 as bait, and mCherry-RZZ or MBP-MAD1/2 as prey. For overnight phosphorylation, bait and prey were incubated with CCC and MPS1^KD^ for 16 hours at 10°C. Before SDS-PAGE analysis, the eluate was dephosphorylated with λ-phosphatase for 30 min at 25°C. Asterisks denote contaminants or degradation products. (**D** and **E**) Quantification of the ROD and MAD1 band intensity normalized to the bait signal from the experiment in (C). Shown are mean and SD from three independent experiments. (**F**) SDS-PAGE analysis of UV-induced cross-linking between BUB1^I471BPA^ and mCherry-RZZ. The asterisk denotes contaminants or degradation products. The experiment was performed three times. (**G**) Scheme for the cell synchronization and RNAi treatment for the experiment in (H). (**H**) Representative images of the localization of Zwilch in stable DLD-1 cell lines expressing different BUB1 constructs treated as indicated in (G). CENP-C was used to visualize kinetochores and DAPI to stain DNA. Scale bar, 5 μm. (**I**) Quantification of Zwilch levels at kinetochores of the experiment shown in (H). *n* refers to individually measured kinetochores and () refers to the number of analyzed cells [left to right: *n* = 2423 (49), 2033 (43), 1152 (33), 1016 (29), 946 (27), and 980 (29)]. The experiment was performed three times. Statistical analysis in (I) was performed with a nonparametric *t* test comparing two unpaired groups (Mann-Whitney test). n.s. *P* > 0.05, ****P ≤ 0.0001. Red bars represent the median (value shown above scatter dot plot) and interquartile range of normalized kinetochore intensities.

In an unanticipated twist, a similar experiment using the same BUB1 bait and RZZ as the prey also revealed robust RZZ binding (Fig. 2, G and H, and fig. S3, B and C). We therefore set out to examine in more detail the molecular basis of this interaction. Within BUB1^437-521^, an N-terminal α helix between residues 465 and 475, coinciding with the ubiquitously conserved central domain 1 (CD1, also called CM1; residues 458 to 476), has been previously shown to interact with the MAD1^CTD^ (*41*, *43*–*47*). AlphaFold (*48*) predicts this N-terminal α helix to be separated by an unstructured intervening region from a second α helix between residues 498 and 507 (Fig. 3B). Despite the low significance of this prediction, residues in this second α helix are only conserved in metazoans, to which RZZ is limited. We generated a series of N-terminal deletion constructs derived from BUB1^437-521^ and tested them in solid-phase binding assays. For unclear reasons, BUB1^467-521^ interacted with RZZ in vitro even more robustly than BUB1^437-521^ (Fig. 2, G and H). Truncation of 10 additional amino acids (BUB1^477-521^) strongly decreased the binding affinity to RZZ, though some residual binding was still observed relative to GST negative control. Any further deletions, including from the C terminus, nearly eliminated RZZ binding (fig. S3, B and C). The robust binding of RZZ to BUB1^467-521^ predicts that BUB1 phosphorylation is dispensable for this interaction. Phosphorylated and unphosphorylated BUB1^437-521^ showed similar binding affinities for RZZ (fig. S5, E and F).

We introduced mutations in GST-BUB1^467-521^ focusing on conserved and exposed residues (Fig. 3, A and B). Mutating Phe^475^ to alanine strongly reduced RZZ (fig. S4, A and B). Mutating Phe^500^ and Glu^501^ also to alanine (467 to 521^FE^) reduced RZZ binding to background levels, as also did combining these mutations with Phe^475^ (467 to 521^FFE^), with or without the inclusion of another mutation, P478R, which had no apparent additional effect on the already near-complete penetrance of the F475A mutation on MAD1:MAD2 or RZZ binding (fig. S4, A and B; this mutant is identified as 467 to 521^FPFE^). Also, in analytical size exclusion chromatography (SEC) experiments, where elution is determined by size and shape, all BUB1 constructs encompassing residues 467 to 521, including GST-BUB1^467-521^ and GST-BUB1^209-521^:BUB3, bound RZZ and coeluted at a reduced elution volume. Conversely, mutants carrying F475A, F500A-E501A, or their combination (with or without P478R) prevented binding in solution (fig. S4, C to H). Despite being paralogous to BUB1:BUB3, BUBR1:BUB3 did not bind RZZ (fig. S4I). Thus, the BUB1 binding site for RZZ is bipartite, with two binding motifs that are both necessary, but individually insufficient, for a robust binding interaction in vitro. Confirming the importance of the first helix for MAD1 binding, both the F475A and the FFE mutants of BUB1^447-521^, which share a dysfunctional first helix, showed little to no binding to MAD1:MAD2. The BUB1 FE mutant, however, had no impact on MAD1:MAD2 interaction, contrary to RZZ binding (Fig. 3, C to E). In line with the crucial role of F475 in both MAD1 and RZZ binding, the previously described BUB1^RRK^ mutant, where F475 is mutated together with two neighboring residues (M474R, F475R, and Q476K) (*43*), reduced both MAD1:MAD2 and RZZ binding to levels similar to those in the negative control (fig. S5, A to D).

Thus, RZZ interacts directly with a segment of BUB1 that is more extended than, but partly overlapping with, the one interacting with the MAD1^CTD^. We used amber codon suppression technology (*49*, *50*) to map the interaction of BUB1 with RZZ. Replacement of the BUB1 residue I471 with the UV-photoactivatable cross-linker *p*-benzoyl-l-phenylalanine (BPA) (Fig. 3A), followed by UV cross-linking, unequivocally indicated that BUB1^467-521^ interacts with ROD (Fig. 3F).

### Validation of the RZZ-binding site of BUB1

To validate the biological significance of these findings, we codepleted endogenous BUB1 and CENP-E (figs. S6, A and B, and S2D) in stable DLD-1 cell lines engineered to express various EGFP-BUB1 fusions, including BUB1^∆467-521^, BUB1^FFE^, BUB1^F475A^, BUB1^FE^, and a construct containing only the BUB3 binding domain (GLEBS motif; BUB1^209-270^) (Fig. 3G and fig. S6C). In absence of transgene expression, RZZ and MAD1 were lost from kinetochores, as expected, whereas the levels of BUBR1 were only partially reduced (Fig. 3, H and I, and fig. S6, H and I). Expression of BUB1^FL^ largely restored RZZ and MAD1 levels at prometaphase kinetochores (Fig. 3, H and I, and fig. S6, D to H). In contrast, expression of BUB1^∆467-521^, BUB1^FFE^, or BUB1^209-270^ in cells depleted of BUB1 and CENP-E completely abrogated kinetochore recruitment of RZZ and MAD1 (Fig. 3, H and I, and fig. S6, D to J), whereas BUB1^F475A^ and BUB1^FE^ had intermediate effects, in line with the biochemical experiments (Fig. 3, H and I, and fig. S6H). Because the kinetochore levels of BUBR1 were fully restored in the presence of BUB1^FFE^ (and of BUB1^F475A^ or BUB1^FE^) (fig. S6I), our observations confirm that BUBR1 cannot replace BUB1 in RZZ or MAD1 recruitment when CENP-E is depleted.

### Two BUB1-dependent axes of corona assembly

**Fig. 4.**
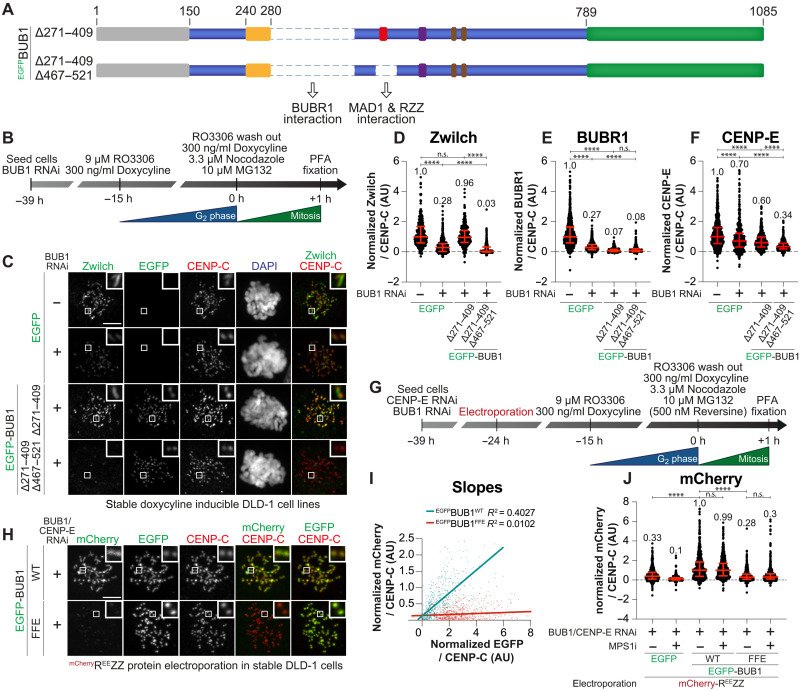
BUB1 and BUBR1 paralogs recruit RZZ to the kinetochore. (**A**) Scheme of DLD-1 lines used in (C). Cells expressed N-terminally EGFP-tagged BUB1 constructs upon the addition of doxycycline. (**B**) Scheme for cell synchronization and RNAi treatment for experiments in (C). (**C**) Representative images showing localization of Zwilch in stable DLD-1 cell lines expressing different BUB1 constructs treated as indicated in (B). CENP-C visualizes kinetochores and DAPI stains DNA. Scale bar, 5 μm. (**D** to **F**) Quantification of Zwilch, BUBR1, and CENP-E at kinetochores for the experiment shown in (C). *n* refers to individually measured kinetochores and () refers to analyzed cells: (D) Left to right: *n* = 304 (23), 614 (31), 362 (24), and 391 (27); (E) left to right: *n* = 809 (33), 825 (35), 246 (19), and 464 (25); (F) left to right: *n* = 3672 (55), 438 (26), 504 (29), and 393 (24). The experiment was performed three times. (**G**) Scheme of cell synchronization and RNAi treatment for experiment in (H). (**H**) Representative images of DLD-1 cell lines electroporated with mCherry-R^EE^ZZ [see (G)]. Scale bar, 5 μm. (**I**) Least-square linear fitting of data points for each kinetochore. mCherry and EGFP intensities are plotted on the *y* and *x* axes for the experiment in (H). (**J**) Quantification of mCherry kinetochore levels for the experiment in (H). *n* and () as above [left to right: *n* = 569 (22), 620 (28), 632 (28), 657 (29), 792 (32), and 658 (30)]. The experiment was performed twice. Statistical analyses in (D) to (F) and (J) were performed with a nonparametric *t* test comparing two unpaired groups (Mann-Whitney test). n.s. *P* > 0.05, ****P ≤ 0.0001. Red bars are median (value shown above scatter dot plot) and interquartile range of normalized kinetochore intensities.

Our studies identify two BUB1-dependent branches for RZZ recruitment, namely, (i) a direct interaction of BUB1^467-521^ with RZZ, and (ii) an interaction of BUB1 with BUBR1 that, in turn, recruits CENP-E for RZZ binding. If correct, this model predicts that a BUB1 mutant impaired in its ability to recruit BUBR1-CENP-E and deleted of the 467 to 521 region that binds RZZ ought to be completely unable to recruit the RZZ. A BUB1 mutant lacking residues 271 to 409 and 467 to 521 (BUB1^∆271-409/467-521^) caused complete elimination of RZZ from kinetochores (Fig. 4, A to F), while deletion of only the BUB1 helix (BUB1^∆271-409^) prevented BUBR1 recruitment but still supported robust RZZ localization (Fig. 4, A to F). Kinetochore CENP-E was reduced in cells expressing BUB1^∆271-409^, and further reduced in cells expressing BUB1^∆271-409/467-521^, in agreement with the idea that BUBR1 and RZZS contribute to CENP-E recruitment.

To verify that the elusive kinetochore receptor of RZZ is indeed the binding site on BUB1 we have identified, we applied our stringent experiment with electroporated mCherry-tagged R^EE^ZZ in cells depleted of endogenous CENP-E and BUB1 and expressing either full-length BUB1^WT^ or BUB1^FFE^ transgenes (Fig. 4G). mCherry-R^EE^ZZ localized robustly to kinetochores in cells expressing BUB1^FL^, but only minimal residual localization was observed in cells expressing BUB1^FFE^ (Fig. 4, H to J). Thus, when CENP-E depletion is coupled with mutations that prevent the interaction of BUB1 with RZZ, even constitutive polymerization in the presence of endogenous MPS1 activity is insufficient for kinetochore recruitment of RZZS. Collectively, these findings provide a compelling recruitment model for RZZ, as further discussed in the Discussion.

### Cooperative binding of MAD1 and the RZZ to BUB1

**Fig. 5.**
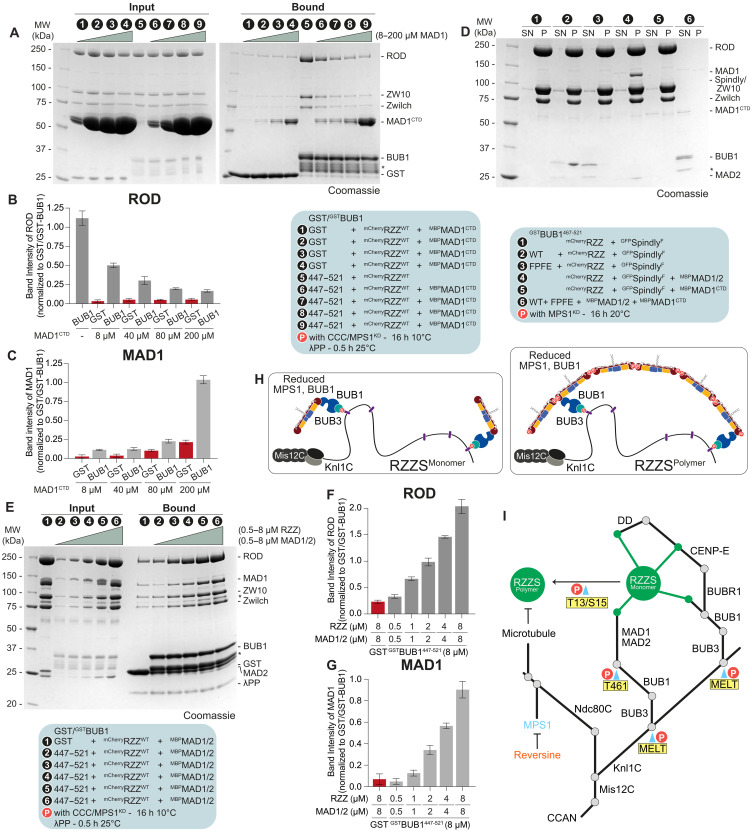
Cooperative binding of MAD1 and the RZZ to BUB1. (**A**) SDS-PAGE analysis of a pulldown assay with either GST or GST-tagged BUB1 as bait, and mCherry-RZZ (concentrations indicated) and MBP-MAD1^CTD^ as prey (concentrations indicated). For overnight phosphorylation, bait and prey were incubated with CCC and MPS1^KD^ for 16 hours at 10°C. The asterisk denotes contaminants or degradation products. (**B** and **C**) Quantification of the ROD and MAD1 band intensity normalized to the bait signal from the experiment depicted in (A). Shown are mean and SD from three independent experiments. (**D**) SDS-PAGE analysis of a pelleting assay. mCherry-RZZ and GFP-Spindly^F^ were polymerized overnight in the presence of MPS1^KD^ at 20°C. Subsequently, polymerized RZZS was incubated with BUB1 or MAD1 constructs for an additional 2 hours at 20°C and lastly applied to a glycerol cushion and spun in an ultracentrifuge. In lane 6, all proteins were mixed in the absence of RZZS to demonstrate lack of pelleting. The asterisk denotes contaminants or degradation products. (**E**) SDS-PAGE analysis of a pulldown assay with either GST or GST-tagged BUB1 as bait, and mCherry-RZZ (concentrations indicated) and MBP-MAD1/2 (monomer concentrations indicated) as prey. For overnight phosphorylation, bait and prey were incubated with CCC and MPS1^KD^ for 16 hours at 10°C. Before SDS-PAGE analysis, the eluate was dephosphorylated with λ-phosphatase for 30 min at 25°C. The asterisks denote contaminants or degradation products. (**F** and **G**) Quantification of the ROD and MAD1 band intensity normalized to the bait signal from the experiment depicted in (E). Shown are mean and SD from three independent experiments. (**H**) Scheme depicting RZZ recruitment to the kinetochore through interaction with BUB1. (**I**) Revised hierarchal organization of outer kinetochore and kinetochore corona based on our results.

An unexpected conundrum of our study, however, concerns the MAD1 recruitment mechanism, as we have shown that the MAD1 and the RZZ binding domains at least partially overlap. This is expected to result in a competition of these proteins for BUB1. To test whether this is the case, we titrated increasing amounts of MAD1^CTD^ into a GST-BUB1 binding assay where RZZ was kept at a constant concentration. In line with our expectation, the amount of RZZ bound to BUB1 progressively decreased as MAD1^CTD^ concentration increased (Fig. 5, A to C). Thus, the MAD1^CTD^ and RZZ bind competitively to an at least partly overlapping region of BUB1. In principle, this problem may be solved by mobilizing a higher proportion of BUB1 than the sum of MAD1 and RZZ, thus buffering the effects of competition. This is a concrete possibility considering the relative cellular concentrations of these species and the fact that KNL1 exposes numerous MELT repeats to increase the multiplicity of BUB1 (*30*, *51*–*57*). Nevertheless, a model purely based on competition seems unsatisfactory, as it would defy previous observations that RZZ facilitates MAD1 recruitment, rather than opposing it (*6*, *26*, *37*, *39*, *41*, *42*, *45*, *58*–*63*). To shed further light on this question, we depleted BUB1 and CENP-E to eliminate MAD1 and RZZS. Reexpression of a BUB1^WT^ transgene largely restored kinetochore MAD1. Albeit at reduced levels, reexpression of BUB1^S459-T461A^, a mutant form of BUB1 that binds RZZ but not MAD1, also restored high levels of kinetochore MAD1 (fig. S7, A to C), in line with the possibility that RZZS contributes to MAD1 kinetochore recruitment downstream of BUB1.

To assess whether RZZ binds MAD1:MAD2 directly, we polymerized RZZS into rings, as described previously (*9*) (fig. S7D) and performed a pelleting assay with BUB1^467-521^ and full-length MAD1:MAD2. MAD1:MAD2 and BUB1^467-521^ pelleted with RZZS, while in the absence of RZZS, both proteins were almost entirely in the supernatant (Fig. 5D). As expected, MAD1^CTD^, which competes with RZZ for BUB1 binding, was instead found in the supernatant (Fig. 5D). In another set of experiments, we titrated increasing amounts of MAD1:MAD2 (0.5 to 8 μM) and RZZ (0.5 to 8 μM) in the presence of GST-BUB1 as bait (8 μM). Rather than competition, we observed a stepwise increase in bound proteins until both components reached apparently equimolar concentrations, in agreement with the idea that, up to approximately stoichiometric concentrations, MAD1:MAD2 and RZZS can bind concomitantly and likely cooperatively in a 3-way complex (Fig. 5, E to G). When MAD1:MAD2 was kept at a fixed concentration, substoichiometric to that of the BUB1 bait, the addition of RZZ led to an initial increase of bound MAD1:MAD2, indicative of cooperative binding to BUB1 and RZZ. Further RZZ additions, however, caused a progressive decrease of MAD1:MAD2 as competition of RZZ on BUB1 started to show its effect (fig. S7, G to I). Collectively, these experiments indicate that despite the apparent competition of the MAD1^CTD^ and RZZ for a partly overlapping binding site on BUB1, a MAD1 binding site on RZZ allows MAD1 to scale with RZZ on the BUB1 bait, provided the latter remains superstoichiometric.

## DISCUSSION

We have dissected the molecular basis of kinetochore recruitment of the kinetochore corona, an essential part of the kinetochore scaffold in early mitosis. The recruitment pathway has its basis in the BUB1:BUBR1 complex of paralogous proteins, whose functional specialization from an original singleton represents a stunning example of molecular evolution (*31*, *33*, *64*). The pathway consists of two branches. The first branch uses BUB1 as a direct receptor through a binding site on ROD. The second branch uses CENP-E, through BUBR1, which docks on BUB1 (*31*, *36*). CENP-E interacts directly with an unknown binding site on the RZZ (*13*). While the two branches seem largely redundant for RZZS localization, we suspect them to be inseparable for proper corona function, as both contribute to robust localization of DD (*65*).

BUB1 lies at the fulcrum of the recruitment pathway. BUB1 is recruited largely through MPS1-dependent phosphorylation of the MELT repeats on KNL1, so that MPS1 inhibition reduces BUB1 kinetochore binding. R^EE^ZZ bypasses the requirement of MPS1 kinase for kinetochore localization when CENP-E has been depleted (*13*). Likely, this is not due to an increase in binding affinity of R^EE^ZZ for BUB1, as RZZ^WT^, R^AA^ZZ (the nonphosphorylatable mutant of RZZ), or R^EE^ZZ bound BUB1 with similar affinity in solid phase or in solution (figs. S4H and S7, E and F). As R^EE^ZZ polymerizes when MPS1 is inhibited, its retention on CENP-E–depleted kinetochores indicates that it sticks more strongly than RZZ monomers to the residual BUB1. This is because monomers may be expected to require at least one corresponding BUB1 receptor for kinetochore recruitment, whereas a multivalent polymer may continue to bind even if only a handful of residual BUB1 remained after MPS1 inhibition (Fig. 5H). Thus, polymerization of the corona provides an extremely robust, cooperative mechanism to remain associated with kinetochores, making it resilient to temporary changes in local phosphorylation patterns and to progressive depletion of binding sites as microtubule attachment proceeds. Residual BUB1 levels due to incomplete depletions, and/or ectopic binding of BUBR1/BUB3 to MELT repeats after depletion of BUB1 (*31*, *33*, *66*), both causing substantial residual RZZ docking, likely explain why previous analyses of the role of BUB1 in RZZ assembly in human cells led to conflicting conclusions (*26*, *36*–*41*). Our observations also argue that BUB1, when bound to RZZ, is unlikely to satisfy a role as a MAD1:MAD2 receptor. We surmise therefore that MAD1:MAD2 complexes recruited to RZZ interact with RZZ-free BUB1 molecules, which likely are numerous given the abundance of MELT repeats on KNL1 (Fig. 5I).

The physical interaction network of the corona integrates SAC and biorientation. By recruiting DD and CENP-E, the corona promotes transport of chromosomes to the spindle equator. By recruiting MAD1:MAD2, the corona promotes SAC signaling. The same mechanisms are also relevant for SAC silencing upon end-on conversion of microtubule attachment. This conversion detaches the chromosome cargo from the corona, activates DD, and allows corona disassembly, removing MAD1:MAD2 from the kinetochore and causing its suppression, which turns off the SAC. Suppression of MPS1 activity, which, in turn, responds to Aurora B kinase, a master regulator of biorientation, is key to SAC silencing. Progressive suppression of MPS1 causes progressive dephosphorylation of the MELT repeats and of MAD1:MAD2 binding to BUB1 and directly inactivates MAD1:MAD2 (*67*, *68*). A crucial unresolved problem for future analyses is therefore how end-on conversion of microtubule attachment changes the inner fabric of interactions within the corona to allow DD activation and the transition to SAC silencing.

## MATERIALS AND METHODS

### Mutagenesis and cloning

The KNL1 constructs (isoform 2; UniProt H0YN41) were subcloned into pCDNA5/FRT/TO-EGFP-IRES as previously described (*69*). The human BUB1 sequence (UniProt O43683) was subcloned into a pCDNA5/FRT/TO-IRES plasmid with an N-terminal EGFP-tag to generate stable cells. The GST-tagged BUB1 truncations, used in vitro, were cloned into a pGEX vector with an N-terminal PreScission-cleavable GST tag. For the ^GST^BUB1^209-521_MBP^/BUB3 construct, the codon-optimized sequence was cloned into a pFLMultiBac vector (*70*). Plasmids for full-length BUB1/BUB3, BUBR1/BUB3, RZZ, Spindly, and MAD1/2 were used as previously described (*9*, *71*–*73*). Mutations and deletions were introduced via site-directed mutagenesis and Gibson assembly (*74*) and verified by Sanger sequencing.

### Expression and purification of RZZ, Spindly, and CCC

The RZZ and Spindly constructs were expressed and purified using the biGBac system (*75*), as previously described (*9*, *11*, *72*). Expression and purification of human CDK1:Cyclin-B:CKS1 (CCC) was carried out as previously described (*76*).

### Expression and purification of ^GST^BUB1 and ^MBP^MAD1^CTD^

The ^GST^BUB1 constructs were expressed in *Escherichia coli* BL21 CodonPlus cells, which were grown in 1 liter of TB at 37°C to an OD_600_ (optical density at 600 nm) of 0.6. The expression was induced with 0.2 mM isopropyl β-D-1-thiogalactopyranoside (IPTG) and the culture was transferred to an incubator precooled to 18°C and grown overnight before harvesting. The pellet was then snap frozen and stored at −80°C until purification. Expression of ^MBP^MAD1^CTD^ (residues 585-C) was carried out in insect cells. The baculovirus was generated in Sf9 cells and used to infect 500 ml of Tnao38 cells, which were grown for 72 hours at 27°C. The pellet was then snap frozen and stored at −80°C until purification. To start the purification, the pellet was resuspended in lysis buffer [50 mM Hepes, pH 8.0, 250 mM NaCl, and 2 mM tris(2-carboxyethyl)phosphine (TCEP)] supplemented with protease inhibitor, 1 mM phenylmethylsulfonyl fluoride (PMSF), and DNase I and lysed by sonication. The lysate was clarified by centrifugation for 45 min at 88,000*g*, sterile filtered, and loaded onto a GSTrap column (Cytiva). Subsequently, the column was washed with at least 20 column volumes of lysis buffer. Elution was performed with lysis buffer supplemented with 25 mM glutathione (GSH). The eluate was and concentrated and loaded onto a Superdex 200 16/60 preequilibrated in SEC buffer (50 mM Hepes, pH 8.0, 250 mM NaCl, and 2 mM TCEP). Peak fractions containing the protein of interest were analyzed by SDS–polyacrylamide gel electrophoresis (SDS-PAGE), concentrated, snap frozen, and stored at −80°C until further usage.

### Expression of BUB1^BPA^

The BUB1 mutant containing the unnatural amino acid BPA was expressed in *E. coli* BL21 strains containing the pEVOLpBpF plasmid (*77*). Cells were cultured in selective TB media, supplemented with 0.2% arabinose, to trigger expression of the tRNA synthetase/tRNA pair and grown at 37°C until an OD_600_ of 0.6 was reached. Expression of BUB1 was induced by adding 0.2 mM IPTG together with BPA at a final concentration of 1 mM. The culture was then transferred into an incubator precooled to 18°C and grown overnight before harvesting. The pellet was then snap frozen and stored at −80°C until purification.

### Expression and purification of ^GST^BUB1^209-521_MBP^/BUB3, BUB1/BUB3, and BUBR1/BUB3

^GST^BUB1^209-521_MBP^/BUB3, BUB1/BUB3, and BUBR1/BUB3 were expressed using the biGBac system as 6×His fusions. The baculovirus was generated in Sf9 cells and used to infect 1 liter of Sf9 cells, which were grown for 72 hours at 27°C. Proteins were immediately purified after harvesting by centrifugation. The pellet was resuspended in lysis buffer (50 mM Hepes, pH 8.0, 300 mM NaCl, 5 mM imidazole, pH 8, 2 mM MgCl_2_, and 2 mM TCEP) supplemented with protease inhibitor, 1 mM PMSF, and DNase I and lysed by sonication. The lysate was clarified by centrifugation for 45 min at 88,000*g*, sterile filtered, and loaded onto a HisTrap HP column (Cytiva). Subsequently, the column was washed with at least 20 column volumes of lysis buffer. The elution was performed with lysis buffer supplemented with 300 mM imidazole, pH 8. The eluate was diluted 1:10 in no salt buffer (50 mM Hepes, pH 8.0, 2 mM MgCl_2_, and 2 mM TCEP), and applied to a 6-ml ResourceQ anion exchange column (Cytiva). The protein was eluted with a 50 to 500 mM NaCl gradient over 10 column volumes and analyzed by SDS-PAGE, and fractions containing the protein of interest were pooled and concentrated with a 100-kDa cutoff Amicon concentrator (Millipore). After three buffer exchanges with SEC buffer (50 mM Hepes, pH 8.0, 150 mM NaCl, 2 mM MgCl_2_, and 1 mM TCEP), the protein was concentrated to the desired concentration, snap frozen, and stored at −80°C until further usage.

### Expression and purification of ^MBP^MAD1/2^FL^

Expression of ^MBP^MAD1/2^FL^ was carried out in insect cells. The baculoviruses were generated in Sf9 cells, and for protein expression, Tnao38 cells were coinfected with ^MBP^MAD1 and MAD2^6His^ cells for 72 hours at 27°C before harvesting. The pellet was washed with phosphate-buffered saline (PBS), snap frozen, and stored at −80°C. For purification, the MAD1/2^FL^ pellet was resuspended in lysis buffer (50 mM Hepes, pH 8.0, 250 mM NaCl, 15 mM imidazole, pH 8, and 1 mM TCEP) supplemented with protease inhibitor, 1 mM PMSF, and DNase I and lysed by sonication. The lysate was clarified by centrifugation for 45 min at 88,000*g*, sterile filtered, and loaded onto a HisTrap HP column (Cytiva). Elution was performed with lysis buffer supplemented with 300 mM imidazole, pH 8. Subsequently, the eluate was diluted 1:10 in zero salt buffer (50 mM Hepes, pH 8.0, and 1 mM TCEP), and applied to a 6-ml ResourceQ anion exchange column (Cytiva). The protein was eluted with a 50 to 500 mM NaCl gradient over 10 column volumes and analyzed by SDS-PAGE, and fractions containing the protein of interest were pooled and concentrated with a 100-kDa cutoff Amicon concentrator (Millipore). After three buffer exchanges with SEC buffer (50 mM Hepes, pH 8.0, 250 mM NaCl, and 1 mM TCEP), the protein was concentrated, snap frozen, and stored at −80°C until further usage.

### Expression and purification of ^mCherry^MPS1^KD^

Expression of the MPS1 kinase domain (KD) was carried out in insect cells as a 6×His fusion protein. The baculovirus was generated in Sf9 cells and used to infect 500 ml of Tnao38 cells, which were grown for 72 hours at 27°C. The pellet was washed with PBS, snap frozen, and stored at −80°C. For purification, the pellet was resuspended in lysis buffer (50 mM Hepes, pH 8.0, 300 mM NaCl, 5% glycerol, 20 mM imidazole, pH 8, and 2 mM TCEP) supplemented with protease inhibitor, 1 mM PMSF, and DNase I and lysed by sonication. The lysate was clarified by centrifugation for 45 min at 88,000*g*, sterile filtered, and loaded onto a HisTrap HP column (Cytiva). Elution was performed with lysis buffer supplemented with 300 mM imidazole, pH 8. The eluate was concentrated and loaded onto a Superdex 200 10/300 preequilibrated in SEC buffer (50 mM Hepes, pH 8.0, 300 mM NaCl, 5% glycerol, and 1 mM TCEP). Peak fractions containing the protein of interest were analyzed by SDS-PAGE, concentrated, snap frozen, and stored at −80°C until further usage.

### Pulldown assays

For pulldown experiments, GST and GST-BUB1 were mixed with preys at the indicated concentrations in 40 μl of binding buffer (50 mM Hepes, pH 8.0, 100 mM NaCl, and 1 mM TCEP) and spun for 30 min at 20,000*g* at 4°C. Where indicated, phosphorylation was carried out by incubating prey and bait for 16 hours at 10°C or for 2 hours at 25°C with ^mCherry^MPS1^KD^ (1:20 kinase-to-substrate ratio) and CCC (1:100 kinase-to-substrate ratio) in binding buffer supplemented with 2 mM ATP and 5 mM MgCl_2_ followed by a centrifugation step for 30 min at 20,000*g* at 4°C. Subsequently, 3 μl of input was taken and mixed with 3 μl of 5× SDS buffer. The remaining 35 μl of the protein mixtures was added to 20 μl of dried GSH beads (preequilibrated in binding buffer) in Pierce microspin columns (Thermo Fischer Scientific) and incubated for 2 hours at 4°C with shaking at 1000 rpm. Next, the incubation step unbound protein was removed by centrifugation (2 min, 800*g*, 4°C) and washed three times with 200 μl of binding buffer with a centrifugating step in-between (2 min, 800*g*, 4°C). Last, the bound fraction was incubated in 20 μl of binding buffer supplemented with 50 mM glutathione, pH 8, for 10 min and eluted by centrifugation (2 min, 1000 g, 4°C). Where indicated, the eluate was treated for 30 min with λ-phosphatase (produced in-house) at 25°C. Last, 10 μl of 5× SDS sample buffer was added to the eluate and analyzed via SDS-PAGE and Coomassie Blue staining or in-gel fluorescence, respectively.

### Analytical SEC

Binding assays in solution were performed under isocratic conditions on a Superose 6 15/50 preequilibrated in SEC buffer (50 mM Hepes, pH 8.0, 100 mM NaCl, and 1 mM TCEP) at 4°C on an ÄKTA pure micro system. The protein absorbance was monitored at 280 nm and the proteins were eluted in 100-μl fractions and analyzed by SDS-PAGE and Coomassie Blue staining. To assess complex formation, proteins were mixed at the indicated concentrations in 60 μl of SEC buffer and incubated for 16 hours at 10°C. Phosphorylation was carried out where indicated in the figure.

### RZZ-Spindly ring formation and pelleting assay

RZZS filaments were formed as described in (*9*). Briefly, 4 μM ^mCherry^RZZ and 8 μM farnesylated ^GFP^Spindly (*72*) were incubated for 16 hours at 20°C in the presence of 1 μM ^mCherry^MPS1^KD^ in 20 μl assay buffer (50 mM Hepes, pH 8, 100 mM NaCl, 2 mM MgCl_2_, 2 mM ATP, and 1 mM TCEP). Subsequently, the preys were added for another 2 hours at 20°C. Next, the protein mixtures were carefully added on 100 μl of glycerol cushion (50 mM Hepes, pH 8, 100 mM NaCl, 2 mM MgCl_2_, 1 mM TCEP, and 40% glycerol) and centrifuged at 100,000*g* for 45 min at 20°C. After spinning, the supernatant was carefully removed and added to 5× SDS buffer, the glycerol cushion was discarded, and the pellet was resuspended in 5× SDS buffer. Last, supernatant and pellet were analyzed by SDS-PAGE followed by Coomassie Blue staining and visualization of the in-gel fluorescence.

### UV cross-linking

For UV cross-linking, the BPA-containing BUB1 mutant was diluted in pulldown buffer (50 mM Hepes, pH 8.0, 100 mM NaCl, and 1 mM TCEP) to a final concentration of 8 μM and mixed with mCherry-RZZ to a final concentration of 2 μM. The samples were incubated for 2 hours at 4°C with shaking at 1000 rpm. Subsequently, the sample was irradiated for 15 min with light-emitting diode UV light at 365 nm wavelength. Successful cross-linking was analyzed via SDS-PAGE.

### Molecular modeling

BUB1 predictions were generated using AlphaFold Multimer (*48*, *78*). Structures and predictions were analyzed using ChimeraX-1.5 (*79*).

### Cell culture and generation of stable cell lines

HeLa and DLD-1 were grown in Dulbecco’s modified Eagle’s medium (DMEM; PAN Biotech) supplemented with 10% tetracycline-free fetal bovine serum (FBS; PAN Biotech) and l-glutamine (PAN Biotech). Cells were grown at 37°C in the presence of 5% CO_2_. Parental Flp-In T-REx DLD-1 osTIR1 cells were a gift from D. C. Cleveland (University of California, San Diego, USA). Stable DLD-1 cell lines were generated using FRT/Flp recombination. KNL1 and BUB1 constructs were subcloned into a pCDNA5/FRT/TO-EGFP-IRES plasmid and cotransfected with pOG44 (Invitrogen), encoding the Flp recombinase using X-tremeGENE (Roche) according to the manufacturer’s instructions. Subsequently, cells were selected for 2 weeks in DMEM supplemented with hygromycin B (250 μg/ml; Thermo Fisher Scientific) and blasticidin (4 μg/ml; Thermo Fisher Scientific). Single-cell colonies were isolated and expanded, and transgene expression was induced through the addition of doxycycline (0.3 μg/ml; Sigma-Aldrich) and checked by immunofluorescence microscopy and immunoblotting.

### RNAi and drug treatment

Depletion of endogenous proteins was achieved through transfection of single small interfering RNA (siRNA) using RNAiMAX (Invitrogen) according to the manufacturer’s instructions. BUB1, CENP-E, and KNL1 were depleted for 36 hours through single transfections. Ndc80C was depleted for 48 hours through two transfections. For a complete list of siRNA oligos used in this study, see table S1. Unless indicated otherwise, nocodazole (Sigma-Aldrich) was used at 3.3 μM, RO3306 (Calbiochem) was used at 9 μM, MG-132 (Calbiochem) was used at 10 μM, and reversine (Cayman Chemical) was used at 500 nM.

### Electroporation of recombinant protein into human cells

Recombinant ^mCherry^RZZ constructs were electroporated as previously described (*80*) using the Neon Transfection System Kit (Thermo Fisher Scientific). HeLa cells were depleted of the indicted proteins as outlined above. Subsequently, cells were trypsinized, washed with PBS, and resuspended in electroporation buffer R (Thermo Fisher Scientific). Recombinant protein was added at a final concentration of 7 μM and electroporated by applying two consecutive 35-ms pulses with an amplitude of 1000 V (HeLa) or 1 pulse for 40 ms with an amplitude of 1400 V (DLD-1). Control cells were electroporated with mCherry or electroporation buffer, respectively. The electroporated sample was subsequently added to 15 ml of prewarmed PBS, centrifuged at 500*g* for 5 min, and subsequently incubated with trypsin/EDTA for 5 min. After two PBS washes, the cell pellet was resuspended in prewarmed DMEM and seeded in a six-well plate with poly-l-lysine–coated coverslips. Following an 8-hour recovery, cells were treated with 9 μM RO3306 (Calbiochem) for 15 hours and doxycycline (300 ng/ml) where indicated. Subsequently, cells were released into mitosis in the presence of 3.3 μM nocodazole and 10 μM MG-132 for 1 hour before fixation for immunofluorescence.

### Immunoblotting

For Western blot analysis, mitotic HeLa and DLD-1 cells were collected via shake-off and resuspended in lysis buffer [150 mM KCl, 75 mM Hepes, pH 7.5, 1.5 mM EGTA, 1.5 mM MgCl_2_, 10% (w/v) glycerol, and 0.075% NP-40] supplemented with protease inhibitor cocktail (Serva) and PhosSTOP phosphatase inhibitors (Roche). After cell lysis, the whole-cell lysates were centrifuged at 22,000*g* for 30 min at 4°C. Subsequently, the supernatant was collected and resuspended in 5× SDS buffer for analysis by SDS–PAGE and Western blotting. The membrane was incubated with the primary antibodies overnight at 4°C diluted in 5% milk-TBS supplemented with 0.1% Tween-20 (TBST). The CENP-E antibody was diluted in TBST without blocking reagent. Subsequently, the nitrocellulose membrane was incubated for 3 hours at 21°C with the secondary antibodies coupled to horseradish peroxidase diluted 1:10,000 in 5% milk-TBST. In-between incubation steps, the membrane was washed three times with TBST for 10 min. After incubation with ECL Western blotting reagent (GE Healthcare), images were acquired with the ChemiDoc MP System (Bio-Rad) using Image Lab 6.0.1 software.

### Immunofluorescence

Immunofluorescence experiments were carried out as previously described (*13*). Briefly, cells were grown on coverslips coated with poly-l-lysine (Sigma-Aldrich). Before fixation, cells were permeabilized with 0.5% Triton X-100 in PHEM (Pipes, Hepes, EGTA, and MgSO_4_) buffer supplemented with 100 nM microcystin for 5 min and fixed with 4% paraformaldehyde (PFA) in PHEM for 20 min. Following fixation, cells were blocked with 5% boiled goat serum (BGS) in PHEM buffer for 1 hour and subsequently incubated for 2 hours at room temperature with the respective primary antibodies in 2.5% BGS-PHEM supplemented with 0.1% Triton X-100. For a complete list of primary antibodies used in this study, see table S2. Subsequently, cells were incubated for 1 hour at room temperature with the respective secondary antibodies (all 1:200 in 2.5% BGS-PHEM supplemented with 0.1% Triton X-100). For a complete list of secondary antibodies used in this study, see table S3. All washing steps were performed three times for 5 min with PHEM supplemented with 0.1% Triton X-100 (PHEM-T) buffer. DNA was stained with 4′,6-diamidino-2-phenylindole (DAPI; 0.5 μg/ml; Serva) and Mowiol (Calbiochem) was used as mounting media.

### Cell imaging

Cells were imaged at room temperature using a spinning disk confocal device on the 3i Marianas system equipped with an Axio Observer Z1 microscope (Zeiss), a CSU-X1 confocal scanner unit (Yokogawa Electric Corporation, Tokyo, Japan), 100×/1.4 numerical aperture oil objectives (Zeiss), and an Orca Flash 4.0 sCMOS Camera (Hamamatsu). Confocal images were acquired as *z*-sections of 0.27 μm (using Slidebook Software 6 from Intelligent Imaging Innovations). Images were converted into maximal intensity projections, converted into 16-bit TIFF files, and exported. Automatic quantification of single kinetochore signals was performed using the software Fiji with background subtraction. Measurements were exported in Excel (Microsoft) and graphed with GraphPad Prism 10 (GraphPad Software). Figures were arranged using Adobe Illustrator 2025.
