# Supplementary material for: The BUB1 and BUBR1 paralogs scaffold the kinetochore fibrous corona
Source: Sci Adv. 2025 Sep 12;11(37):eady6890. doi: 10.1126/sciadv.ady6890 (PMC12429063; doi:10.1126/sciadv.ady6890)
Supplement: Supplementary file 1 — Figs. S1 to S7 Tables S1 to S3 [file sciadv.ady6890_sm.pdf]

Science Advances
AAAS

Supplementary Materials for

The BUB1 and BUBR1 paralogs scaffold the kinetochore fibrous corona

Verena Cmentowski and Andrea Musacchio

Corresponding author: Verena Cmentowski, verena.cmentowski@mpi-dortmund.mpg.de;
Andrea Musacchio, andrea.musacchio@mpi-dortmund.mpg.de

Sci. Adv. **11**, eady6890 (2025)
DOI: 10.1126/sciadv.ady6890

This PDF file includes:

Figs. S1 to S7
Tables S1 to S3

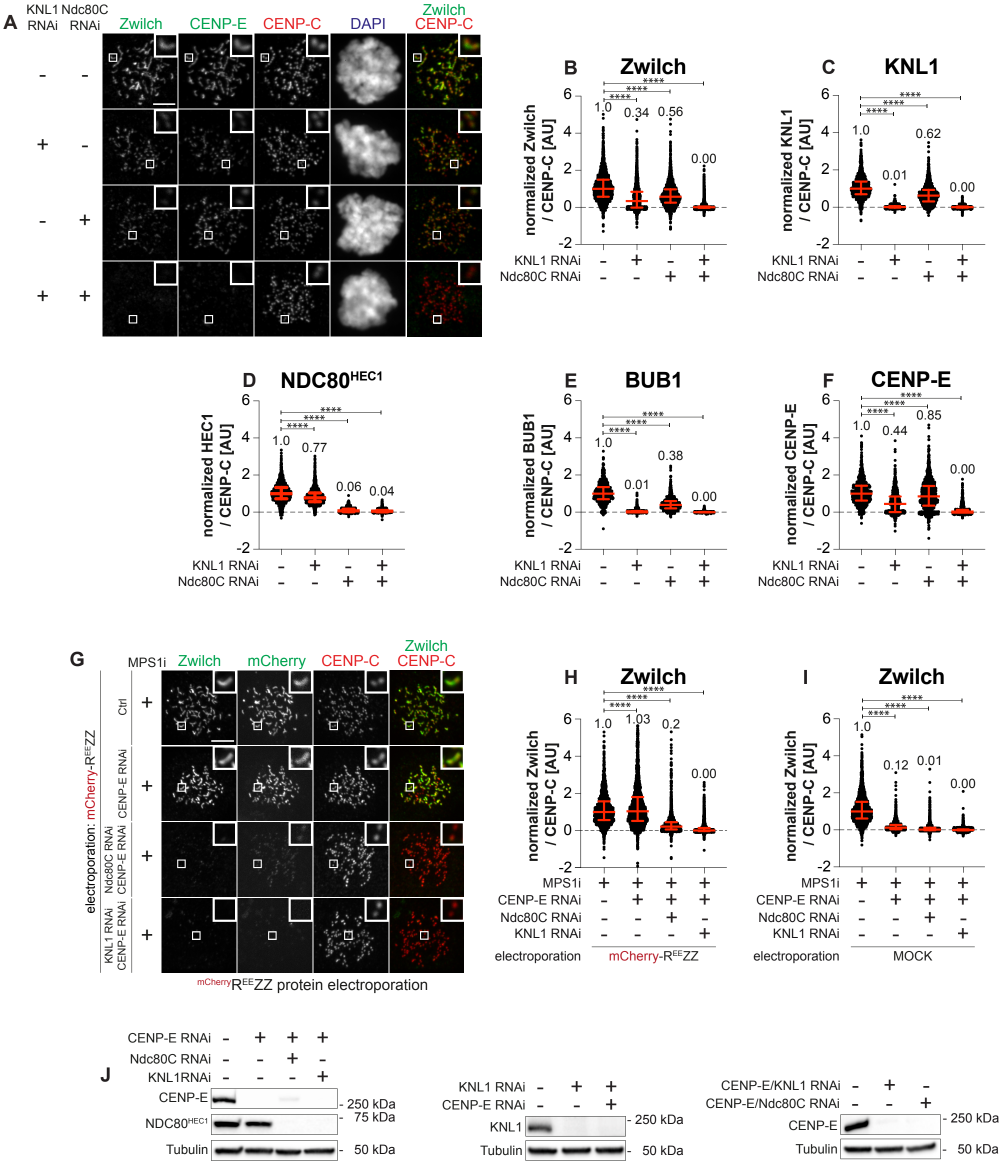

Supplement 1

Fig. S1. (**A**) Representative images of HeLa cells after RNAi treatment to deplete KNL1 and/or Ndc80C. Ndc80C RNAi treatment has been performed for 48 h. KNL1 RNAi has been performed for 40 h. 8 h after the second transfection with siRNA to deplete Ndc80C, cells were synchronized in G2 phase with RO3306 for 15 h and then released into mitosis. Subsequently, cells were immediately treated with 3.3 µM nocodazole and 10 µM MG132 for an additional hour. CENP- C was used as a kinetochore marker and DAPI to visualize DNA. Scale bar: 5 µm (**B-F**) Quantification of Zwilch, KNL1, NDC80^{HEC1}, BUB1 and CENP-E levels at kinetochores of the experiment shown in (A). n refers to individually measured kinetochores and () to number of

analyzed cells: (B) left to right: n=2369 (43), 2557 (45), 2670 (50), 3381 (59), (C) left to right: n=1781 (43), 1475 (37), 1212 (32), 1156 (34), (D) left to right: n=1781 (43), 1475 (37), 1212 (32), 1156 (34), (E) left to right: 638 (22), 999 (33), 815 (28), 649 (23), (F) left to right: n=899 (27), 1065 (27), 1278 (29), 1371 (31). The experiment has been performed three times. (**G**) Representative images showing the localization of Zwilch in cells electroporated with mCherry-R^{EE}ZZ treated with the respective RNAi as indicated in the figure according to the scheme shown in **Fig. 1D**. CENP-C was used to visualize kinetochores. Scale bar: 5 µm (**H-I**) Quantification of Zwilch levels at kinetochores of the experiment shown in (G). n refers to individually measured kinetochores and () to number of analyzed cells: (H) left to right: n=1806 (37), 3078 (45), 2113 (15), 2342 (47), (I) left to right: n=2544 (35), 3000 (41), 2529 (37), 1792 (34). The experiment has been performed three times. (**J**) Immunoblot of mitotic HeLa cells showing the depletion efficiency of the proteins indicted in the figure. 50 µg of cleared lysate was used for each condition, and Tubulin is shown as a loading control.

Statistical analyses in panels B-F and H-I were performed with a nonparametric t-test comparing two unpaired groups (Mann–Whitney test). Symbols indicate: n.s. $p > 0.05$, $^{*}p \leq 0.05$, $^{**}p \leq 0.01$, $^{***}p \leq 0.001$, $^{****}p \leq 0.0001$. Red bars represent the median (value shown above scatter dot plot) and interquartile range of normalized kinetochore intensity values.

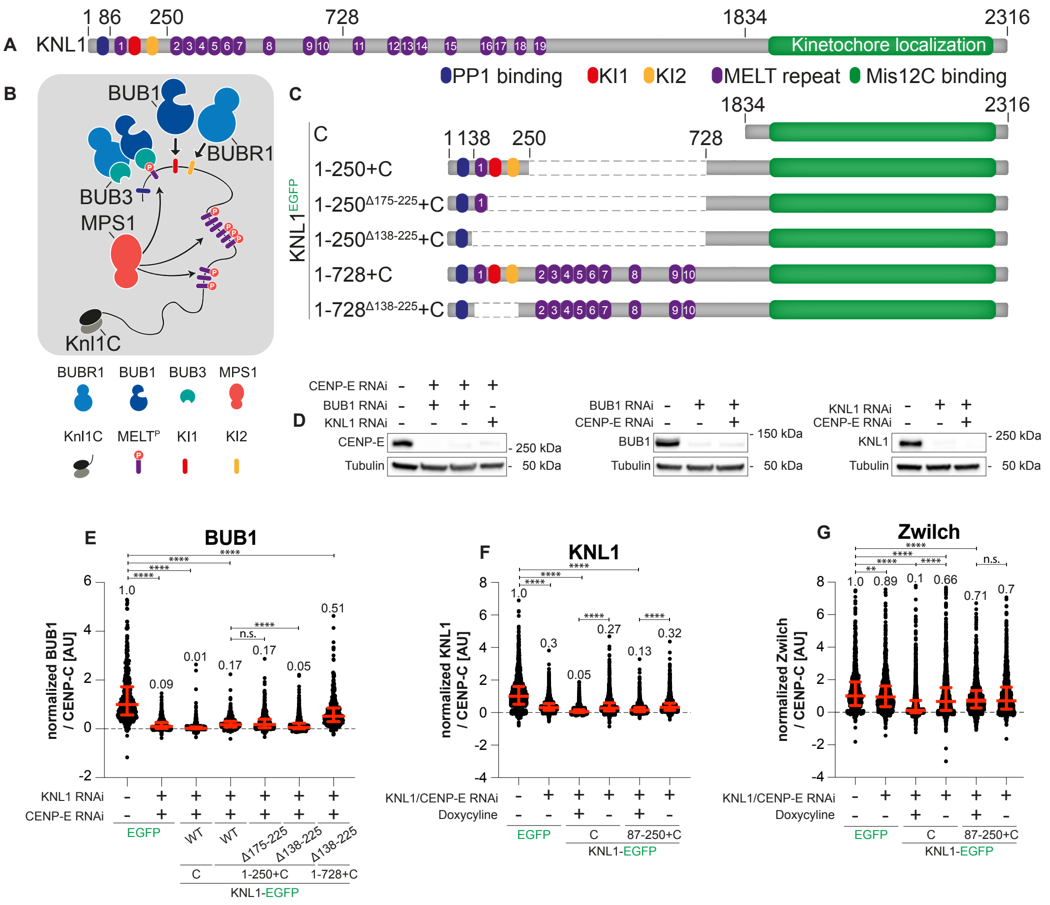

Supplement 2

Fig. S2. (**A**) Schematic representation of the organization of KNL1 with relevant functional domains. (**B**) Schematic depiction of the KNL mediated recruitment of BUB1:BUB3 and BUBR1:BUB3 through phosphorylated MELT repeats and the KI motifs. (**C**) Schematic of the stable DLD-1 cell lines used in the experiment depicted in **Fig. 1H**. Cells express C-terminally EGFP-tagged KNL1 constructs upon addition of doxycycline. (**D**) Immunoblot of mitotic DLD-1 cells showing the depletion efficiency of the proteins indicted in the figure. 50 µg of cleared lysate was used for each condition, and Tubulin is shown as a loading control. (**E**) Quantification of BUB1 levels at kinetochores of the experiment shown in **Fig. 1H**. n refers to individually measured kinetochores and () to number of analyzed cells (left to right: n=500 (29), 823 (33), 520 (22), 514 (23), 609 (28), 537 (22), 446 (18)). The experiment has been performed three times. (**F-G**) Quantification of KNL1 and Zwilch levels at kinetochores of stable DLD-1 cell lines expressing different KNL1 constructs. Cells were depleted of KNL1 and CENP-E under suboptimal silencing conditions by shortening the depletion duration from 36 h (**Fig. S2D**) to 24 h. 16 h before fixation protein expression was induced through addition of doxycycline and cells were synchronized in G2 phase with RO3306 for 15 h. Subsequently, cells were released into mitosis and immediately

treated with 3.3 μM nocodazole, 10 μM MG132 and doxycycline for one hour before fixation. n refers to individually measured kinetochores and () to number of analyzed cells: (F) left to right: n=1695 (41), 2409 (50), 2366 (50), 2096 (47), 1600 (39), 2248 (48), (G) left to right: n=850 (25), 777 (23), 1575 (40), 1485 (37), 955 (28), 1094 (29). The experiment has been performed three times.

Statistical analyses in panels E-G were performed with a nonparametric t-test comparing two unpaired groups (Mann–Whitney test). Symbols indicate: n.s. $p > 0.05$, *$p \leq 0.05$, **$p \leq 0.01$, ***$p \leq 0.001$, ****$p \leq 0.0001$. Red bars represent the median (value shown above scatter dot plot) and interquartile range of normalized kinetochore intensity values.

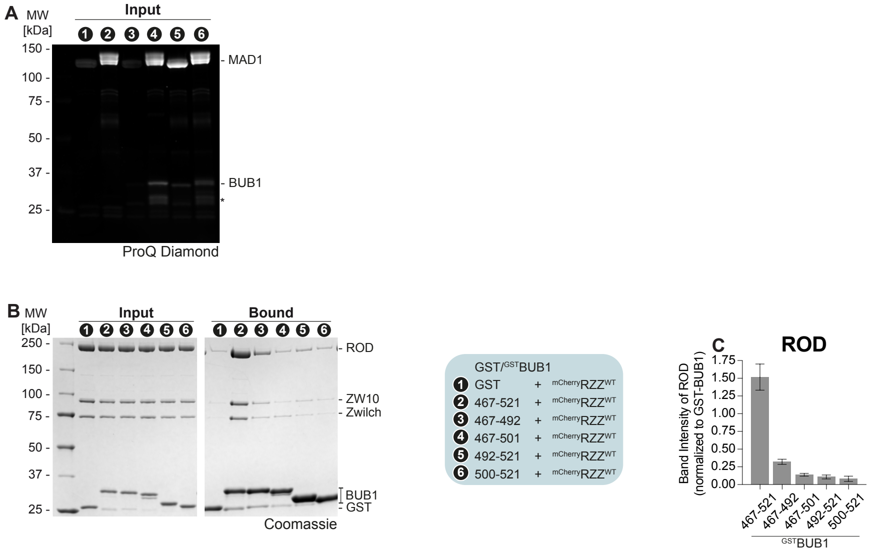

Supplement 3

Fig. S3. (**A**) ProQ Diamond staining was used to verify the successful phosphorylation of bait and prey from the experiment shown in **Fig. 2E**. The asterisk denotes contaminants or degradation products. (**B**) SDS-PAGE analysis of a pulldown assay with either GST or GST-tagged BUB1 as bait, and mCherry-RZZ as prey. (**C**) Quantification of the ROD band intensity normalized to the bait signal from the experiment depicted in (B). Shown are mean and standard deviation from three independent experiments.

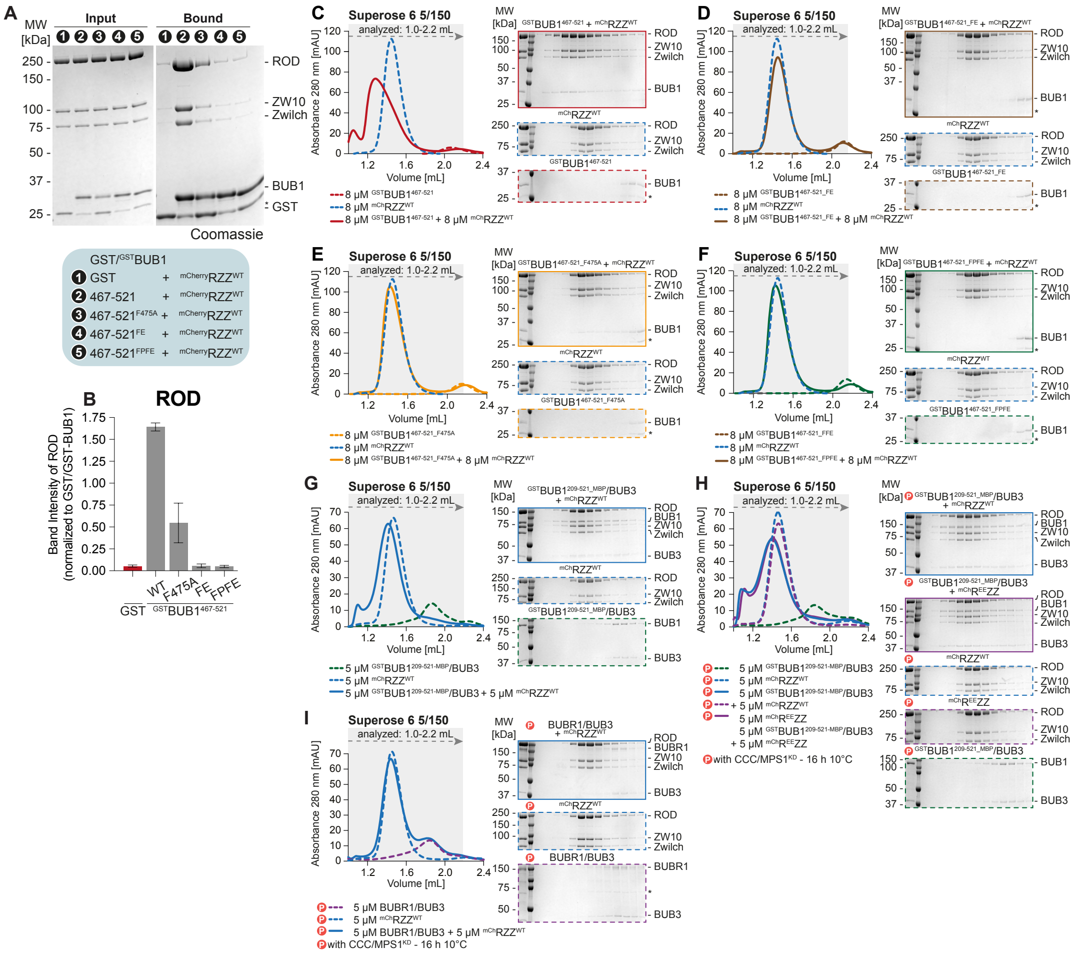

Supplement 4

Fig. S4. (**A**) SDS-PAGE analysis of a pulldown assay with either GST or GST-tagged BUB1 as bait and mCherry-RZZ as prey. The asterisk denotes contaminants or degradation products. (**B**) Quantification of the ROD band intensity normalized to the bait signal from the experiment depicted in (A). Shown are mean and standard deviation from three independent experiments. (**C-I**) Analytical SEC binding assays with GST-BUB1 constructs, mCherry-RZZ constructs, and BUBR1/BUB3. The run with combined species is represented as a continuous line and runs of individual species with a dashed line. The control gels with mCherry-RZZ alone are shared between panels (C-F). For overnight phosphorylation bait and prey were incubated with CCC and MPS1^{KD} for 16 h at 10°C. The asterisks denote contaminants or degradation products.

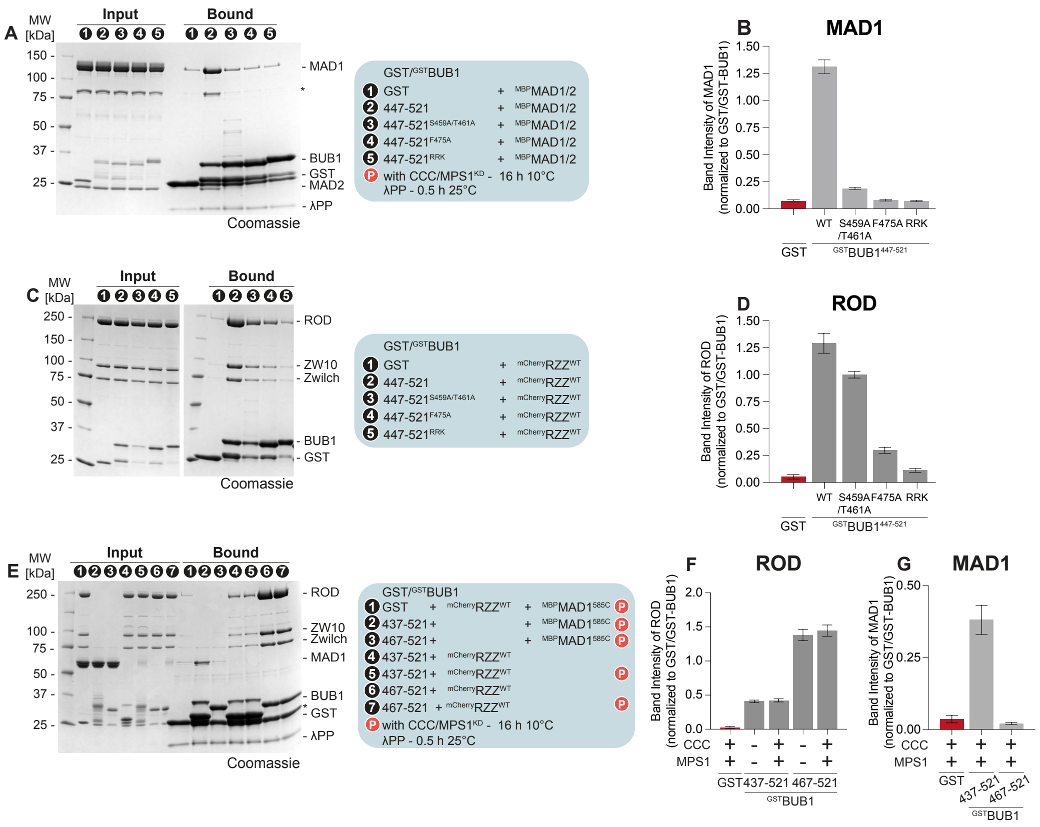

Supplement 5

Fig. S5. (**A**) SDS-PAGE analysis of a pulldown assay with either GST or GST-tagged BUB1 as bait and MBP-MAD1/2 as prey. For overnight phosphorylation bait and prey were incubated with CCC and MPS1^{KD} for 16 h at 10°C. Before SDS-PAGE analysis the eluate was dephosphorylated with λ-phosphatase for 30 mins at 25°C. The asterisk denotes contaminants or degradation products. (**B**) Quantification of the MAD1 band intensity normalized to the bait signal from the experiment depicted in (A). Shown are mean and standard deviation from three independent experiments. (**C**) SDS-PAGE analysis of a pulldown assay with either GST or GST-tagged BUB1 as bait and mCherry-RZZ as prey. (**D**) Quantification of the ROD band intensity normalized to the bait signal from the experiment depicted in (C). Shown are mean and standard deviation from three independent experiments. (**E**) SDS-PAGE analysis of a pulldown assay with either GST or GST-tagged BUB1 as bait and mCherry-RZZ or MBP-MAD1/2 as prey. For overnight phosphorylation bait and prey were incubated with CCC and MPS1^{KD} for 16 h at 10°C. Before SDS-PAGE analysis the eluate was dephosphorylated with λ-phosphatase for 30 mins at 25°C. The asterisk denotes contaminants or degradation products. (**F-G**) Quantification of the ROD and MAD1 band intensity normalized to the bait signal from the experiment depicted in (E). Shown are mean and standard deviation from three independent experiments.

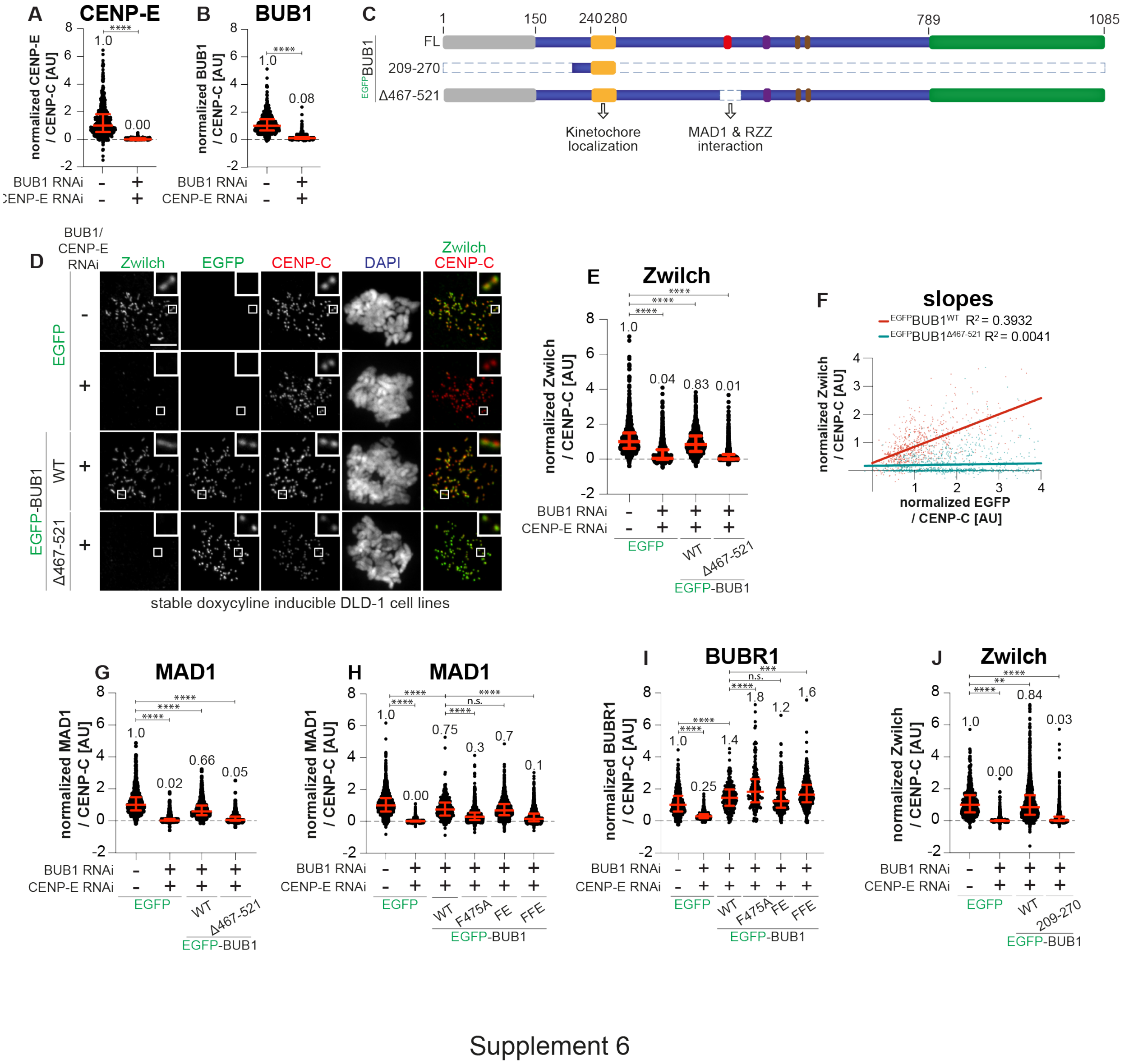

Fig. S6. (**A-B**) Quantification of CENP-E and BUB1 levels at kinetochores after depletion of the indicated proteins. n refers to individually measured kinetochores and () to number of analyzed cells: (A) left to right: n=633 (25), 892 (33), (B) left to right: n=734 (27), 819 (31). The experiment has been performed three times. (**C**) Schematic of the stable DLD-1 cell lines used in the experiment depicted in (D). Cells express N-terminally EGFP-tagged BUB1 constructs upon addition of doxycycline. (**D**) Representative images showing the localization of Zwilch in stable DLD-1 cell lines expressing different BUB1 constructs treated as indicated in **Fig. 3G**. CENP-C was used to visualize kinetochores and DAPI to stain DNA. Scale bar: 5 µm. (**E**) Quantification of Zwilch levels at kinetochores of the experiment shown in (D). n refers to individually measured kinetochores and () to number of analyzed cells (left to right: n=1032 (33), 1171 (40), 640 (26),

1142 (34)). The experiment has been performed three times. (**F**) Least-square linear fitting of data points for each kinetochore of the Zwilch intensity on the y-axis and the EGFP intensity on the x-axis of the experiment shown in (D). (**G**) Quantification of MAD1 levels at kinetochores of the experiment shown in (D). n refers to individually measured kinetochores and () to number of analyzed cells (left to right: n=833 (27), 913 (30), 749 (28), 987 (31)). The experiment has been performed three times. (**H-I)** Quantification of MAD1 and BUBR1 levels at kinetochores of the experiment shown in **Fig. 3H**. n refers to individually measured kinetochores and () to number of analyzed cells: (H) left to right: n=1882 (40), 1375 (36), 345 (18), 1408 (36), 1353 (36), 2080 (42), (I) left to right: n=467 (22), 399 (18), 209 (16), 181 (15), 385 (17), 413 (20). The experiment has been performed three times. (**J**) Quantification of Zwilch levels at kinetochores of stable DLD-1 cells treated as indicated in **Fig. 3G**. n refers to individually measured kinetochores and () to number of analyzed cells (left to right: n=679 (21), 512 (19), 1343 (29), 963 (25)). The experiment has been performed three times.

Statistical analyses in panels A-B, E and G-J were performed with a nonparametric t-test comparing two unpaired groups (Mann–Whitney test). Symbols indicate: n.s. $p > 0.05$, *$p \leq 0.05$, **$p \leq 0.01$, ***$p \leq 0.001$, ****$p \leq 0.0001$. Red bars represent the median (value shown above scatter dot plot) and interquartile range of normalized kinetochore intensity values.

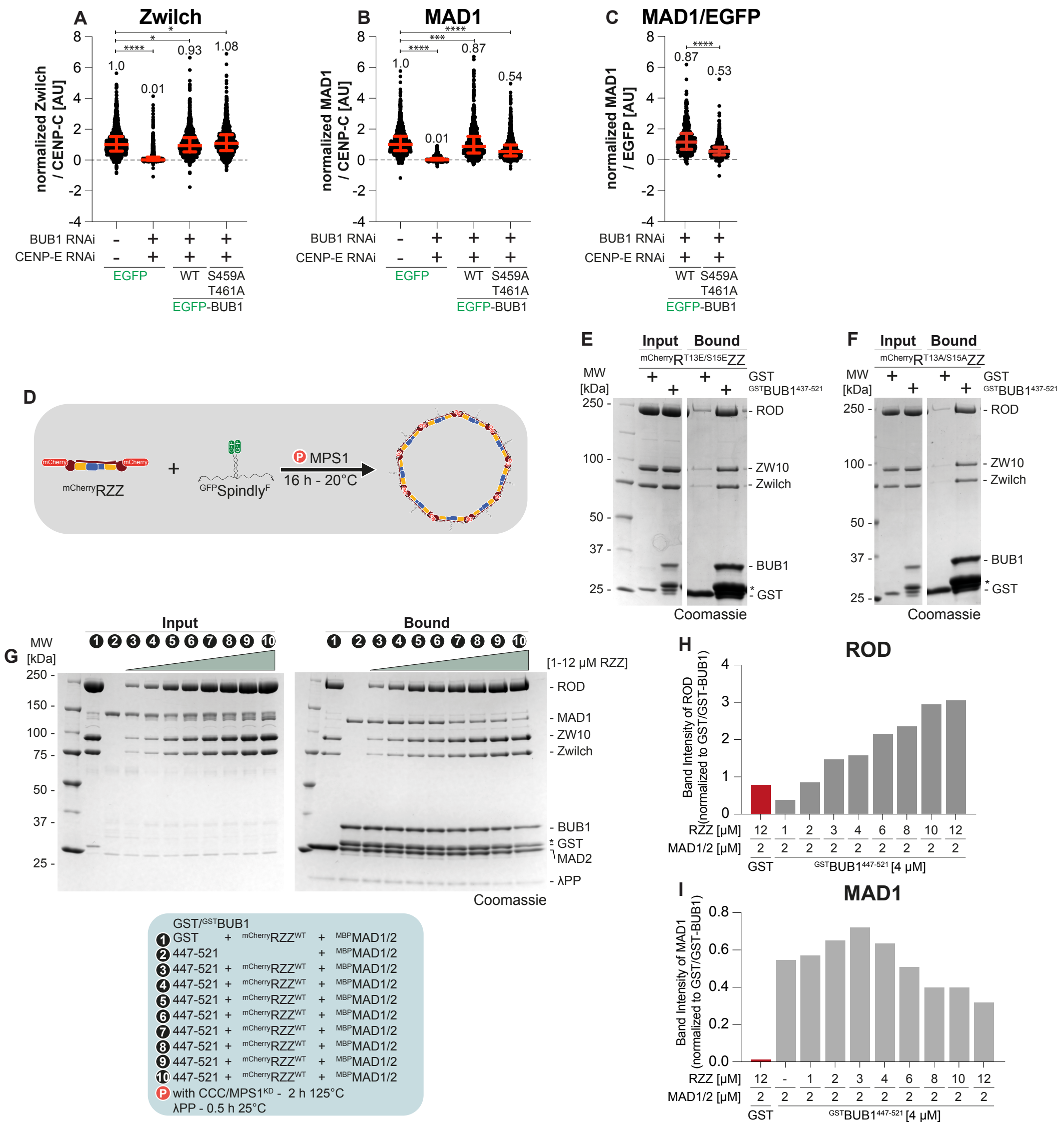

Supplement 7

Fig. S7. (**A-B**) Quantification of Zwilch and MAD1 levels at kinetochores of stable DLD-1 cells treated as indicated in **Fig. 3G**. n refers to individually measured kinetochores and () to number of analyzed cells: (A) left to right: n=1445 (39), 1059 (33), 1028 (32), 1012 (33), (B) left to right: n=1424 (39), 786 (29), 944 (34), 1145 (36). The experiment has been performed two times. (**C**) Ratio of the MAD1/EGFP levels at kinetochores of the experiment quantified in (A-B). n refers to individually measured kinetochores and () to number of analyzed cells (left to right: n=944 (34), 1145 (36)). (**D**) Schematic of RZZS ring formation experiment used in the experiment depicted **Fig. 5D**. (**E-F**) SDS-PAGE analysis of a pulldown assay with either GST or GST-tagged BUB1$^{437-521}$ as bait, and different mCherry-RZZ constructs as prey. The asterisks denote contaminants or degradation products. The experiment has been performed three times. (**G**) SDS-PAGE analysis

of a pulldown assay with either GST or GST-tagged BUB1 as bait, mCherry-RZZ (concentrations indicated) and MBP-MAD1/2 (monomer concentrations indicated) as prey. For overnight phosphorylation, bait and prey were incubated with CCC and MPS1^{KD} for 16 h at 10°C. The asterisk denotes contaminants or degradation products. (**H-I**) Quantification of the ROD and MAD1 band intensity normalized to the bait signal from the experiment depicted in (G).

Table S1.

List of the siRNA oligos used in this study.

Target mRNA	Sequence (3'-5')	Concentration	Source
BUB1	Dharmacon: GGUUGCCAACACAAGUUCU	50 nM	Krenn *et al.*, 2014 (69)
CENP-E	Dharmacon: AAGGCUACAAUGGUACUAUAU	60 nM	Ciossani *et al.*, 2018 (42)
KNL1	Invitrogen:<br>HSS183683: CACCCAGUGUCAUACAGCCAAUAUU<br>HSS125942: UCUACUGUGGUGGAGUUCUUGAUAA<br>HSS125943: CCCUCUGGAGGAAUGGUCUAAUAAU	60 nM	Krenn *et al.*, 2014 (69)
Ndc80C	Sigma-Aldrich:<br>siHEC1: GAGUAGAACUAGAAUGUGA<br>siSPC24: GGACACGACAGUCACAAUC<br>siSPC25: CUACAAGGAUUCCAUCAAA	20 nM	Polley *et al.*, 2024 (17)

Table S2.
List of the primary antibodies used in this study.

Epitope	Species	Dilution	Manufacturer (cat. number)	Identifier
BUB1	Rabbit polyclonal	1:500 (IF)<br>1:5000 (IB)	Abcam, #ab9000	RRID: AB_940664<br>discontinued
BUBR1	Rabbit polyclonal	1:1000 (IF)	Thermo Scientific #720297	RRID: AB_2610165<br>discontinued
CENP-C	Guinea pig polyclonal	1:1000 (IF)	MBL, #PD030	RRID: AB_10693556<br>discontinued
CENP-E	Rabbit monoclonal	1:200 (IF)<br>1:500 (IB)	Abcam, #ab133583	RRID: AB_2910100
HEC1	Mouse monoclonal	1:1000 (IF/IB)	Abcam, #ab3613	RRID: AB_303949
KNL1	Rabbit polyclonal	1:750 (IF)<br>1:500 (IB)	made in-house, #SI0787	N/A
MAD1- DyLight488	Mouse monoclonal	1:200 (IF)	made in-house, Clone BB3-8	N/A
MAD1-DyLight550	Mouse monoclonal	1:200 (IF)	made in-house, Clone BB3-8	N/A
α-tubulin	Mouse monoclonal	1:8000 (IB)	Sigma-Aldrich, #T9026	RRID: AB_477593
Zwilch	Rabbit polyclonal	1:750 (IF)	made in-house, #SI520	N/A

Table S3.

List of the secondary antibodies used in this study.

Name	Species	Manufacturer (cat. number)	Identifier
α-mouse Alexa Fluor 488	Goat	Invitrogen A11001	RRID: AB_2534069
α-mouse Rhodamine Red	Goat	Jackson Immuno Research 115-295-003	RRID: AB_2338756
α-rabbit Alexa Fluor 488	Donkey	Invitrogen A21206	RRID: AB_2535792
α-rabbit Rhodamine Red	Donkey	Jackson Immuno Research 711-295-152	RRID: AB_2340613
α-guinea pig Alexa Fluor 647	Goat	Invitrogen A-21450	RRID: AB_141882
α-mouse HRP	Sheep	Amersham, NXA931-1ML	RRID: AB_772209
α-rabbit HRP	Donkey	Cytiva (GE), NA934V	RRID: AB_772206
